# A Universal Mechanism Ties Genotype to Phenotype in Trinucleotide Diseases

**DOI:** 10.1371/journal.pcbi.0030235

**Published:** 2007-11-23

**Authors:** Shai Kaplan, Shalev Itzkovitz, Ehud Shapiro

**Affiliations:** 1 Department of Biological Chemistry, Weizmann Institute of Science, Rehovot, Israel; 2 Department of Molecular Cell Biology, Weizmann Institute of Science, Rehovot, Israel; 3 Department of Computer Science and Applied Mathematics, Weizmann Institute of Science, Rehovot, Israel; Washington University, United States of America

## Abstract

Trinucleotide hereditary diseases such as Huntington disease and Friedreich ataxia are cureless diseases associated with inheriting an abnormally large number of DNA trinucleotide repeats in a gene. The genes associated with different diseases are unrelated and harbor a trinucleotide repeat in different functional regions; therefore, it is striking that many of these diseases have similar correlations between their genotype, namely the number of inherited repeats and age of onset and progression phenotype. These correlations remain unexplained despite more than a decade of research. Although mechanisms have been proposed for several trinucleotide diseases, none of the proposals, being disease-specific, can account for the commonalities among these diseases. Here, we propose a universal mechanism in which length-dependent somatic repeat expansion occurs during the patient's lifetime toward a pathological threshold. Our mechanism uniformly explains for the first time to our knowledge the genotype–phenotype correlations common to trinucleotide disease and is well-supported by both experimental and clinical data. In addition, mathematical analysis of the mechanism provides simple explanations to a wide range of phenomena such as the exponential decrease of the age-of-onset curve, similar onset but faster progression in patients with Huntington disease with homozygous versus heterozygous mutation, and correlation of age of onset with length of the short allele but not with the long allele in Friedreich ataxia. If our proposed universal mechanism proves to be the core component of the actual mechanisms of specific trinucleotide diseases, it would open the search for a uniform treatment for all these diseases, possibly by delaying the somatic expansion process.

## Author Summary

Trinucleotide diseases are a broad family of hereditary diseases characterized genetically by an expanded DNA region consisting of a repeated three-letter code. Patients inheriting such an abnormal DNA region experience sudden disease onset at an age that inversely depends on the size of the expanded region, followed by inevitable and highly predictable suffering and death. Despite more than a decade of research, the underlying mechanism of these diseases remains an enigma. Although the genes implicated with the various trinucleotide diseases are unrelated, and the defects in these genes occur in different parts of the DNA coding for the gene, the diseases' shared characteristics suggest a common mechanism underlies their root cause. We suggest a mechanism that uniformly explains how the inherited DNA repeats genetically encode the time of onset and the rate of progression of trinucleotide diseases. It suggests the disease manifests and progresses through the further expansion of the inherited abnormally expanded DNA region. It explains the clinical data of many diseases in this family, including previously unexplained onset-related phenomena. It also predicts that a general therapy for these diseases would be a drug or procedure that successfully interferes with the ongoing expansion of the disease trinucleotide repeat.

## Introduction

Trinucleotide diseases are hereditary disorders in which a gene that harbors a trinucleotide repeat is inherited with a number of repeats that exceeds a disease-specific threshold [1,2]. In the so-called polyglutamine diseases, including Huntington disease (HD) [3], spinocerebellar ataxia (SCA) of various types [4], and others, the expanded repeat CAG codes for glutamine in a gene's coding region. Polyglutamine diseases are manifested by neuronal symptoms [1]. In other diseases, the repeat is located in noncoding regions: in the muscle disease myotonic dystrophy type 1 (DM1) [1,5] the CTG repeat is located in the 3′ untranslated region (UTR) of the gene *DMPK*, and in Friedreich ataxia [1,5,6] (FRDA) a GAA repeat is located within the first intron of the gene *FRDA.*

The genes associated with the various diseases are structurally and functionally unrelated. Despite their differences, many of the trinucleotide diseases share intriguing phenotype characteristics [2,7]. The disease has no symptoms for many years until a sudden onset at an age that is inversely correlated with the number of inherited repeats [2,4,8–11]. For example, in HD, the median onset age may change from 67 y for patients with 39 repeats to 27 y for patients with 50 repeats [11]. When the number of repeats exceeds 70, the disease has juvenile onset; there are also cases of childhood onset for even longer repeats [12,13]. These relations of onset age and the number of repeats are similar in other diseases, and are typically characterized by an exponential curve in which the change in the age of onset as a result of additional inherited repeat reduces with the number of repeats [4,8,14]. A larger number of repeats also directly correlates with the severity and the rate of symptom progression of the disease [12,15,16]. In addition, many diseases show genetic anticipation, where the number of inherited repeats increases significantly from generation to generation, usually via paternal transmission, thus causing earlier onset and faster progression [1,2,7].

The mechanism, which leads to such genetically encoded delay in disease onset, is yet unknown. For polyglutamine diseases, it is currently assumed that the extended polyglutamine has a gain of a toxic function which leads to cumulative damage in the affected cells, possibly in the form of glutamine aggregate formation [1,17–19]. It is assumed that the level of toxicity depends on the number of repeats, such that longer repeats are more toxic and lead to a faster damage and earlier cell death, implying that both disease and delay in onset are governed by the same mechanism [1,17–19].

This suggested mechanism of cumulative damage has several shortcomings and is unlikely to explain the strong correlations of onset and repeat length. First, the strong correlations of repeat length and age of onset are also apparent in nonpolyglutamine diseases such as DM1 and FRDA, suggesting a mechanism that is unrelated to the specific gene function or expression level. Second, in the rare case of patients with homozygous mutation (two expanded alleles), the cumulative damage mechanism would predict a significant decrease in age of onset, which is in contradiction with recent clinical findings that homozygousity does not result in earlier onset [20–22]. Unlike onset, disease progression after onset was found to be notably faster in homozygote patients with HD, leading to the suggestion that two different mechanisms account for the delayed onset and the disease pathology [20]. Furthermore, aggregate accumulation mechanism is highly sensitive to differences in expression level and thus unlikely to show such precise correlations. Finally, it is unclear how such a mechanism would result in the exponential onset curve often seen in polyglutamine diseases.

Several previous studies in trinucleotide diseases animal models, including mouse [23–25] and fruit fly [26], have highlighted the fact that trinucleotide repeats present significant somatic instability, which is specifically significant in the disease-affected tissues. Somatic length instability was also shown in lymphoblastoid cell lines of HD subjects with intermediate length [27]. This somatic instability is a result of either slippage mutation or a mishybridization of the two DNA strands due to the high complementarities of the repeating sequence followed by a DNA repair process [2,26,28]. This process was shown to have strong bias toward expansion [23–26]. The repeat instability increases with the number of repeats [29,30], as the likelihood of mishybridization grows with repeat length. Thus, as the disease allele somatically expands within a cell, its probability for further expansion increases, leading to an accelerated expansion process.

Understanding the mechanism by which the number of inherited repeats affects the onset age and disease progression is highly desirable, as it may open new treatment opportunities. Here, we propose that a universal mechanism of length-dependent somatic mutation underlies trinucleotide diseases and accounts for these striking genotype–phenotype correlations.

## Results

### The Mechanism

**Figure 1:**
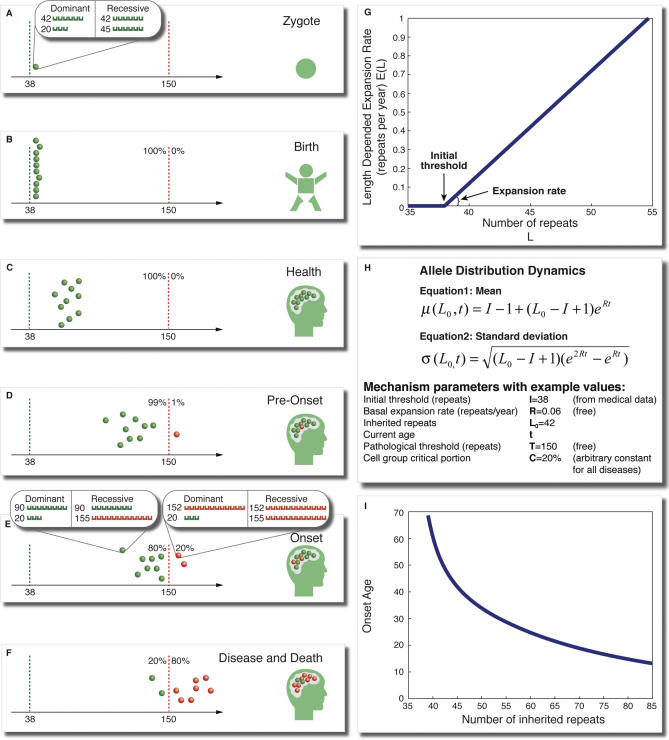
A Universal Mechanism for Trinucleotide Diseases (A) The patient inherits one gene (or two genes in recessive disease) that harbours a trinucleotide repeat that exceeds disease-specific threshold (green line, *I* = 38 repeats in this example). (B) A disease-specific group of cells that determines the disease onset and progression is initially clustered around the inherited value. (C) During the lifetime of the patient, the number of repeats in these cells increases stochastically, (D) some crossing a pathological threshold (red line, 150 in this example) while the patient is still considered healthy. (E) Disease commences when in a critical portion of these cells (*C* = 20% in this example) the number of repeats crosses the pathological threshold. (F) The disease progresses toward death as more cells cross the target threshold. (G) The rate of allele expansion *E* at any given time is a linearly increasing function of the number of repeats above the initial threshold. (H) Equations for the mean and standard deviation of allele size as a function of the patient's age *t*, inherited number of repeats *L_0_*, and the mechanism parameters (see Materials and Methods and Text S1). (I) The mechanism predicts an exponentially decreasing onset curve similar to curves obtained from clinical data for trinucleotide diseases.

**Figure 2:**
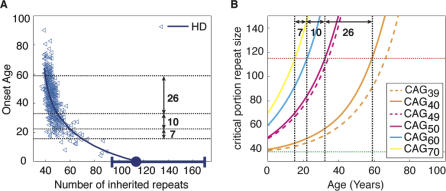
Exponential Onset Curve (A) The mechanism, with parameters fitted to the clinical data [8] of HD, predicts pathological threshold around 115 CAG repeats. (B) The somatic expansion of repeats as a function of age of patients with HD with various inherited allele size. Onset occurs when enough cells (critical portion of 20%) cross the pathological threshold (red line). The slower expansion of shorter alleles (40–50 repeats) accounts for a larger difference in the age of onset (26 y) in contrast to longer alleles (60–70 accounts for only 7 y). A single-repeat (39–40) difference in short alleles close to the initial threshold (green line) may reduce several years from onset age compared to a single repeat difference in longer alleles (49–50).

**Figure 3:**
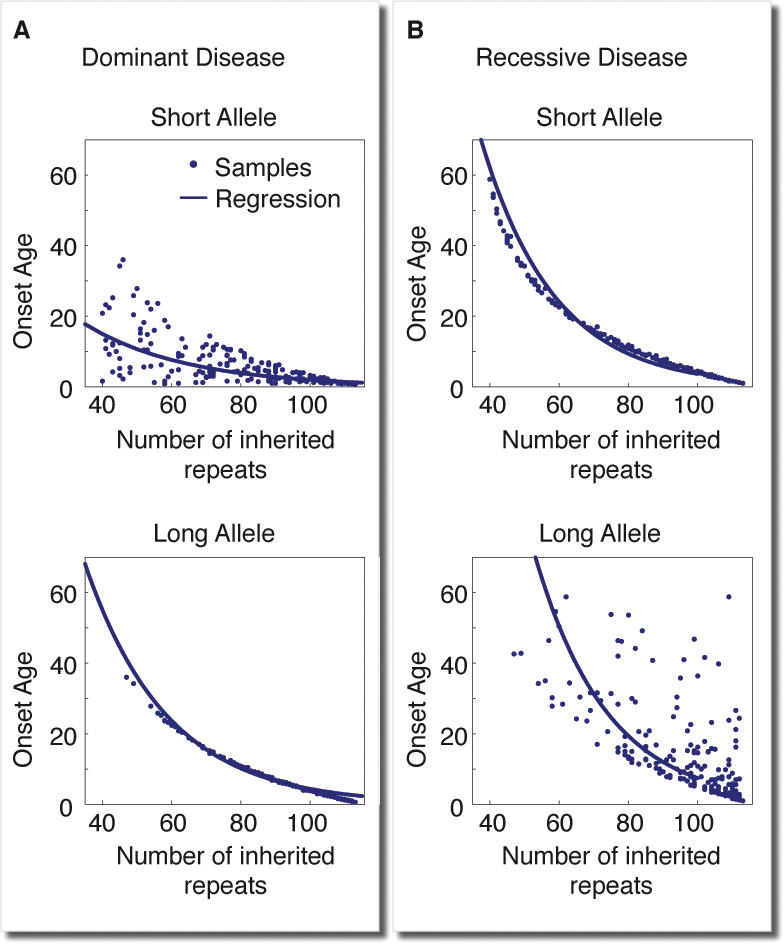
Computer Simulations of the Mechanism in Patients with Two Disease Alleles Simulation results for patients of (A) dominant and (B) recessive diseases with various combinations of two inherited alleles. The age of onset as a function of short/long allele size and regression line are presented. In a dominant disease (A), only the longer allele size is in strong anticorrelation (*r* = −0.99) with age of onset, while in a recessive disease only the shorter allele is in strong anticorrelation (*r* = −0.99), consistently with corresponding data for dominant polyglutamine diseases and the recessive disease FRDA [32].

**Figure 4:**
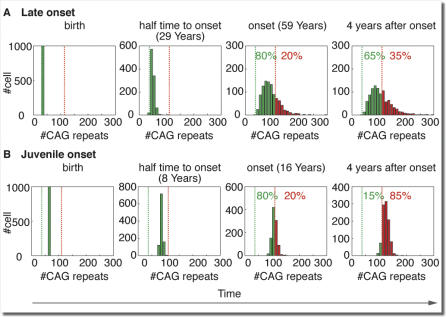
Simulation of Two Hypothetical Patients with HD The variability of repeat-size distribution is zero at birth and increases during the patient's lifetime as a result of the independent stochastic expansion process. At the time of onset (20% of the cells exceed the pathological threshold) a wide distribution in the CAG_40_ patient with late onset (A) accounts for slower progression (only 35% of cells exceed pathological threshold after 4 y) while a narrow distribution in the CAG_70_ patient with juvenile onset accounts for faster progression (85% after 4 y).

Our proposed mechanism specifies that onset and progression of the disease are determined by the rate of expansion of the trinucleotide repeat in certain cells in the patient's body. The disease manifests when the trinuecleotide repeat has expanded beyond a certain threshold in a sufficient number of these cells, and progresses as more and more cells do so. For each disease, our universal mechanism, described in Figure 1, assumes that a patient inherits the disease gene in which one allele (if the disease is dominant, or two alleles if it is recessive) has a trinucleotide repeat larger than the disease-specific initial threshold, and predicts that: (1) the patient has a disease-specific group of cells, the dynamics of which determines the onset age and progression rate of the disease (Figure 1A–F); (2) the disease alleles of cells in this group stochastically expand at a rate that increases linearly with the number of repeats (Figure 1G); (3) when the number of repeats in one allele (if it is dominant; two if recessive) is larger than a disease-specific pathological threshold, the cell enters a disease-specific pathological state (Figure 1D); (4) disease onset occurs when a critical portion of the cells in the group has entered the pathological state (Figure 1E); and (5) the disease progresses in severity, toward death, as more cells enter the pathological state (Figure 1F). We studied the dynamics of the mechanism and its implications on various disease-related properties using computer simulations and a mathematical analytical model (see Materials and Methods and Figure 1H).

### Exponential Onset Curve

We have conducted computer simulations and mathematical analysis of our proposed mechanism (see Materials and Methods and Text S1) and used them to compute the expected age of onset for patients with various inherited repeat lengths. Our results show that such a process leads to an exponentially decreasing age of onset curve typically seen in clinical data of trinucleotide diseases. Furthermore, by fitting our model parameters to previously published clinical data of each disease (see Figures 2A and S1), we can estimate both the length of the pathological threshold assumed by our mechanism and the rate of somatic trinucleotide expansion (the initial threshold of each disease can be accurately determined from the clinical data). Figure 2A shows the onset curve predicted by the mechanism, with mechanism parameters fitted to Huntington clinical data [8]. The predicted pathological threshold for HD is 115 repeats (see Text S1). Figure 2B demonstrates how the trinucleotide repeats of patients with HD with various inherited repeat lengths are predicted by our mechanism to expand exponentially during the patient's lifetime toward the pathological threshold, leading to the observed onset age differences. The slow expansion rate associated with smaller number of inherited repeats eventually leads to a large change in the onset age as a result of a single difference in the number of inherited repeats as seen in the clinical data.

### The Recessive Trinucleotide Disease FRDA

While most trinucleotide diseases are autosomal dominant, FRDA is the only known autosomal recessive trinucleotide disease. In this disease, the repeat sequence GAA is found in the first intron of the gene coding for Frataxin. A patient with FRDA has inherited two expanded disease alleles, which typically range in size from 200 repeats and up to more than 1,000 repeats. Previous studies [10,31,32] of FRDA showed that onset age is in strong correlation with the size of the shorter allele but not with the longer allele size or with the average size of both alleles. A mechanism based on a slow accumulation of toxicity cannot account for this unique phenomenon, as both alleles contribute to the level of Frataxin in the cell. In contrast, our mechanism of somatic expansion toward a pathological threshold provides a simple explanation. The long allele somatically expands beyond the pathological threshold earlier, as it is not only inherited with a number of repeats closer to that threshold but also starts with a faster expansion rate. Being a recessive disease, the cell enters its pathological state only when the shorter allele also expands beyond this threshold. Computer simulations of our mechanism in patients with various size combinations of two alleles (see Figure 3) demonstrate that in a recessive disease, onset age is in strong correlation with the size of the short allele. In contrast, our mechanism predicts that in patients of dominant diseases with two diseased alleles (so-called homozygous patient), onset age correlates with the size of the longer allele, as reported previously [33] (see Figure 3).

### Correlations with Disease Progression

In trinucleotide diseases there is also a correlation between the number of repeats and the rate of symptoms progression [12,15,16]. A patient with HD with more than 70 CAG repeats may manifest a juvenile onset before the age of 20 y and a much more aggressive course of disease progression compared to a late-onset patient with 40 inherited CAG repeats. Our proposed mechanism provides a simple explanation to this difference in progression rate. At birth, all disease-related cells in a patient's body carry the inherited allele size, and the repeat length variability between cells is negligible. However, this variability grows during the patient's lifetime as the trinucleotide repeat in each cell in this group expands independently and stochastically. Disease onset occurs when enough cells enter the pathological state by expanding beyond the pathological threshold, and the disease progresses as more and more cells enter the pathological state. A wide repeat size distribution near the time of onset implies that many of the cells are still far from the pathological threshold and hence accounts for a slow progression rate. In contrast, a narrow distribution near the onset time implies that many cells are about to exceed the pathological threshold leading to a fast progression rate (see Figure 4 and Videos S1 and S2). Computer simulations of the length-dependent expansion process quantify the effect of the number of inherited repeats on disease progression. In these simulations, we arbitrarily defined disease onset to be the time when 20% of the cells have entered a pathological state and calculated the time until 80% of the cells enter a pathological state (shorter time indicates faster progression). The results (see Figure 4) show that the progression is much slower for late-onset patients (CAG_40_) than for patients with juvenile onset (CAG_70_). Since all patients are born with negligible variability in the size of the trinucleotide repeat, the shorter time to onset and the fast expansion in the juvenile case leads to a smaller variability near the time of onset and thus accounts for the faster progression.

### Onset and Progression in Patients with Homozygous Mutation

**Figure 5:**
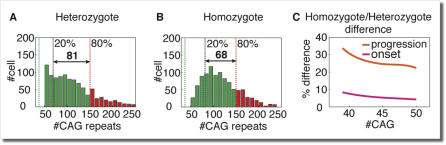
Homozygote versus Heterozygote Patients (A–C) Simulation results show the long allele distribution at onset for heterozygote patients (A) with onset at 60 y and homozygote patients (B) with onset at 56 y, both with 40 inherited repeats. The homozygote patients show more narrow distribution, which is closer to the pathological threshold, which leads to a faster disease progression. (C) The difference in age of onset is rather small (homozygote ∼6% earlier) and therefore is undetectable considering other variability factors; however, the difference in progression is significant (homozygote ∼30% faster).

In rare cases, patients with polyglutamine diseases carry two copies of the disease allele and are considered homozygote to the disease. One would expect that if polyglutamine toxicity damage accumulated from the patient's birth time, having two copies of a disease allele would have a tremendous effect on the age of onset. However, recent clinical studies of homozygote patients did not find any reduction in the expected age of onset due to homozygousity [20–22]. On the other hand, the rate of progression was significantly higher in HD homozygote patients compared to nonhomozygote patients. The fact that homozygousity increases the rate of progression but has no effect on age of onset cannot be explained only by toxicity or aggregate accumulation, and requires additional explanations. Our proposed mechanism provides one of the first explanations for this puzzling phenomenon. According to this mechanism in dominant diseases, a cell in the disease-related group of cells enters a pathological state when the first of its two alleles has expanded beyond the pathological threshold. We have conducted computer simulations of the somatic expansion mechanism that compares the longer allele size distribution in a patient homozygous for the disease (extreme value distribution of the two-allele sizes) with the long allele of a patient with heterozygous mutation. The simulations show that the distributions at the time of onset are nearly similar for the most expanded alleles (see Figure 5A and 5B), which accounts for the similar onset age. However, as we go toward the least expanded alleles in the distribution, the homozygote distribution is narrower and closer to the pathological threshold, explaining the faster progression. Simulation of patients with various allele sizes (see Figure 5C) show that the reduction in onset age in the homozygote case is minor (∼6%), while the change in the disease progression is significant (∼30%).

### Supporting Evidence from Other Experimental and Clinical Data

Mouse models of trinucleotide diseases demonstrate that somatic mutations exist in the disease-associated tissue and that those mutations expand with age [23–25]. If indeed mouse and human disease-related biochemistry is similar at the cellular level, our mechanism predicts that for a mouse to show disease symptoms during its short lifespan, it must be born with a disease allele very close to the pathological threshold (115 in HD according to our prediction). Indeed, mouse models of polyglutamine diseases typically require a number of repeats larger than 100 in order to show disease symptoms [34,35]. The creation of symptomatic mouse models with a number of repeats similar to that of human failed despite efforts to significantly increase the expression of the diseased gene [36,37]. This is consistent with our mechanism and in addition suggests that toxicity of short repeats cannot be increased by higher expression and that pathology is only seen when the repeat is much longer than typical inherited human genotype. In contrast to patients with HD, knock-in HD mice homozygous for HD mutation show anticipated age of onset compared to heterozygotes in addition to a more progressive disease [38]. This further supports our prediction that the mouse models that manifest HD symptoms are born with a number of repeats larger than, or very close to, the pathological threshold. In such a case, our model indeed predicts that homozygosity would have a stronger effect on onset age.

Clinical studies [39–41] show that if an inherited CAG repeat is interrupted by another trinucleotide sequence, age of onset is delayed significantly compared to patients without such an interrupt. For example, a patient with SCA type 1 (SCA1) who had a CAG_58_ repeat with an interrupt of a CAT repeat after 45 repeats had an onset age of 50 y rather than the expected onset age of 22 y. Other studies of DNA repeats showed that such an interrupt significantly slows the repeat's rate of mutation [29,30]. In addition, it was shown that the rate of mutation of tandem repeats depend on the length of the pure uninterrupted repeat segment [29,30] (45 in the above case); thus, our mechanism, which is based on the rate of somatic mutation, accurately predicts the observed change in onset age resulting from the above interrupt location.

## Discussion

We suggest that a length-dependent somatic expansion mechanism underlies the genetically encoded delayed onset of trinucleotide diseases. According to the mechanism, the inherited disease allele has no toxic implications on the disease-related cells before it expands beyond a disease-specific pathological threshold, leading to cell pathology. Several clinical and experimental findings provide support for this mechanism. First, it provides a simple explanation to the correlation between age of onset and number of inherited repeats uniformly for both polyglutamine and nonpolyglutamine diseases. In addition, the disease dynamics implied by our mechanism explains the exponential shape of the onset curves, the faster progression associated with juvenile onset, the correlation with the short allele only in the recessive disease FRDA, and the similar onset but faster progression for patients with HD with homozygous mutations. The commonly assumed mechanism of cumulative damage or slow aggregate formation does not seem to be able to explain most of these disease-related phenomena. Our mechanism does not contradict studies in mouse models, which are focused on understanding the pathology of the different diseases, showing that this pathology occurs only when the number of repeats is sufficiently long. Thus, it provides explanation to the large repeat number that is required for symptomatic mouse models.

The universal mechanism suggested in this work may apply to many trinucleotide diseases. Nevertheless, it provides several predictions that may be subject to further experimental validation in a disease-specific context, possibly by the use of animal models. One challenge is to identify for each disease which group of cells triggers disease onset. Our mechanism predicts that the somatic expansion in this group of cells would be particularly high. Another prediction is that somatic repeat expansion is expected to progress with the age of an affected animal even prior to disease onset. Finally, the model predicts that the rate of repeat expansion increases with time, and that at any time is a function of the repeat length at that time. Newly available technologies that facilitate the amplification and measurement of the repeat length at a single-cell resolution may characterize and accurately measure the mutation progress rate for various cell populations in the affected organ of mouse models for various diseases.

Our mechanism suggests that the disease gene is not toxic for many years and that the time to onset is counted by a silent expansion of the repeat with no physiological implication on the cell. This may have significant clinical implications on the effort to find therapies for these cureless inherited diseases. Rather than addressing direct causes of pathology such as polyglutamine aggregates, therapeutic effort may focus on delaying the onset by slowing the somatic expansion process, which is known to be mediated by DNA repair mechanisms [26,28]. Our mechanism predicts that an ability to slow this expansion process may provide a common therapy to patients of most trinucleotide disease, as it addresses the universal component of the mechanism rather than the disease-specific component.

## Materials and Methods

### Model parameters.

*Disease-specific parameters. I*—the initial threshold. A patient that inherits allele with number of repeats longer than this threshold will have the disease during his lifetime. *T*—the pathological threshold. Cells, which their alleles have somatically expanded beyond this threshold, become pathological. In the recessive version of the model both allele needs to expand beyond this threshold to become pathological. *R*—the basal expansion rate. This parameter determines the contribution of single additional repeat to the length dependent rate of expansion. *C*—the critical portion. The portion of pathological cells that is required for the disease onset.

*Patient parameters. t*—the patient's age. *L_0_*—the patient's number of inherited repeats.

### Computer simulations.

Computer simulations were performed on a group of 1,000 cells in which the number of repeats was initialized to *L*_0_, the number of inherited repeats. At each time point *t*, the number of repeats in each cell, *L_t_*, was expanded based on its value at the beginning of the simulation time unit. The size of expansion was taken from a Poisson distribution with expectance *E* (*L_t_*) = (*L_t_* − *I*) × *R* where *E* is the expected expansion per time unit based on the cell current allele size *L_t_*, the initial threshold *I*, and the rate of mutation *R*. The simulation sampling rate, 5 y^−1^, is much larger than the typical mutation rate, 0.05 y^−1^, and thus is sufficient for accuracy. The critical portion of cohort size *C* = 20% was used as a threshold for onset, although other choices for *C* ( *C* = 5%–50%) gave qualitatively similar results.

### Simulations of two alleles.

To simulate recessive disease and compare it with dominant disease patient with two disease alleles (Figure 3), we simulated the two alleles independently for all combinations of two allele sizes between 39 and 50 using the disease parameter from the example in Figure 1 (*I* = 38, *T* = 150, *R* = 0.06). The onset was determined according to the model, and the correlations with the small and large alleles were measured. The qualitative results hold for any disease-specific parameter set. The same simulation parameters were used to create the distribution of homozygote versus heterozygote patients. The percent difference in onset *O* and duration *D* was calculated as follows:

Duration Difference = 100 × (*D_Heteroozygote_* − *D_Homozygote_*) / *D_Heteroozygote_*

   Onset Difference = 100 × (*O_Heteroozygote_* − *O_Homozygote_*) / *O_Heteroozygote_*

### Analytical model.

We have derived an analytical model that describes the dynamic behavior of the mean and the standard deviation of allele size distribution that is stochastically expanding under the length-dependent expansion rate assumed by the mechanism we describe. The equations (shown in Figure 1) derived by the model were used to fit clinical data of the various diseases (see Text S1). Detailed derivation of the model equations is described in Text S1.

## Supporting Information

Figure S1 Model Parameter Fitting to Onset Age Clinical Datasets of Various Trinucleotide Diseases (326 KB JPG)

Text S1 Detailed Derivation of the Model Equations and Model Parameter Fitting (410 KB DOC)

Video S1 Simulation of Allele Size Distribution Dynamics in Patients with Late Onset (40 Repeats) (1.8 MB QT).

Video S2 Simulation of Allele Size Distribution Dynamics in Patients with Juvenile Onset (70 Repeats) (1.9 MB QT).

### Accession Numbers

The Entrez Gene (http://www.ncbi.nlm.nih.gov/sites/entrez?db=gene) accession numbers for the genes discussed in this paper are *HD* (3064), *FRDA* (2395), *SCA1* (6310), *SCA2* (6311), *SCA3* (4287), *SCA6* (773), *SCA7* (6314), *DM1* (1760), and *DRPLA* (1822).

## Abbreviations

FRDA: Friedrich ataxia
HD: Huntington disease

## References

[pcbi-0030235-b001] Gatchel JR, Zoghbi HY (2005). Diseases of unstable repeat expansion: Mechanisms and common principles. Nat Rev Genet.

[pcbi-0030235-b002] Pearson CE, Nichol Edamura K, Cleary JD (2005). Repeat instability: Mechanisms of dynamic mutations. Nat Rev Genet.

[pcbi-0030235-b003] Bates GP (2005). History of genetic disease: The molecular genetics of Huntington disease—A history. Nat Rev Genet.

[pcbi-0030235-b004] Schols L, Bauer P, Schmidt T, Schulte T, Riess O (2004). Autosomal dominant cerebellar ataxias: Clinical features, genetics, and pathogenesis. Lancet Neurol.

[pcbi-0030235-b005] Ranum LP, Day JW (2004). Pathogenic RNA repeats: An expanding role in genetic disease. Trends Genet.

[pcbi-0030235-b006] Alper G, Narayanan V (2003). Friedreich's ataxia. Pediatr Neurol.

[pcbi-0030235-b007] Everett CM, Wood NW (2004). Trinucleotide repeats and neurodegenerative disease. Brain.

[pcbi-0030235-b008] Squitieri F, Ciarmiello A, Di Donato S, Frati L (2006). The search for cerebral biomarkers of Huntington's disease: A review of genetic models of age at onset prediction. Eur J Neurol.

[pcbi-0030235-b009] Antonini G, Giubilei F, Mammarella A, Amicucci P, Fiorelli M (2000). Natural history of cardiac involvement in myotonic dystrophy: Correlation with CTG repeats. Neurology.

[pcbi-0030235-b010] Mateo I, Llorca J, Volpini V, Corral J, Berciano J (2003). GAA expansion size and age at onset of Friedreich's ataxia. Neurology.

[pcbi-0030235-b011] Brinkman RR, Mezei MM, Theilmann J, Almqvist E, Hayden MR (1997). The likelihood of being affected with Huntington disease by a particular age, for a specific CAG size. Am J Hum Genet.

[pcbi-0030235-b012] Squitieri F, Cannella M, Simonelli M (2002). CAG mutation effect on rate of progression in Huntington's disease. Neurol Sci.

[pcbi-0030235-b013] Squitieri F, Pustorino G, Cannella M, Toscano A, Maglione V (2003). Highly disabling cerebellar presentation in Huntington disease. Eur J Neurol.

[pcbi-0030235-b014] David G, Durr A, Stevanin G, Cancel G, Abbas N (1998). Molecular and clinical correlations in autosomal dominant cerebellar ataxia with progressive macular dystrophy (SCA7). Hum Mol Genet.

[pcbi-0030235-b015] Rosenblatt A, Liang KY, Zhou H, Abbott MH, Gourley LM (2006). The association of CAG repeat length with clinical progression in Huntington disease. Neurology.

[pcbi-0030235-b016] Illarioshkin SN, Igarashi S, Onodera O, Markova ED, Nikolskaya NN (1994). Trinucleotide repeat length and rate of progression of Huntington's disease. Ann Neurol.

[pcbi-0030235-b017] Perutz MF, Windle AH (2001). Cause of neural death in neurodegenerative diseases attributable to expansion of glutamine repeats. Nature.

[pcbi-0030235-b018] Bennett MJ, Huey-Tubman KE, Herr AB, West AP, Ross SA (2002). Inaugural article: A linear lattice model for polyglutamine in CAG-expansion diseases. Proc Natl Acad Sci U S A.

[pcbi-0030235-b019] Clarke G, Lumsden CJ, McInnes RR (2001). Inherited neurodegenerative diseases: The one-hit model of neurodegeneration. Hum Mol Genet.

[pcbi-0030235-b020] Squitieri F, Gellera C, Cannella M, Mariotti C, Cislaghi G (2003). Homozygosity for CAG mutation in Huntington disease is associated with a more severe clinical course. Brain.

[pcbi-0030235-b021] Toyoshima Y, Yamada M, Onodera O, Shimohata M, Inenaga C (2004). SCA17 homozygote showing Huntington's disease-like phenotype. Ann Neurol.

[pcbi-0030235-b022] Narain Y, Wyttenbach A, Rankin J, Furlong RA, Rubinsztein DC (1999). A molecular investigation of true dominance in Huntington's disease. J Med Genet.

[pcbi-0030235-b023] Kennedy L, Shelbourne PF (2000). Dramatic mutation instability in HD mouse striatum: Does polyglutamine load contribute to cell-specific vulnerability in Huntington's disease?. Hum Mol Genet.

[pcbi-0030235-b024] Kennedy L, Evans E, Chen CM, Craven L, Detloff PJ (2003). Dramatic tissue-specific mutation length increases are an early molecular event in Huntington disease pathogenesis. Hum Mol Genet.

[pcbi-0030235-b025] Fortune MT, Vassilopoulos C, Coolbaugh MI, Siciliano MJ, Monckton DG (2000). Dramatic, expansion-biased, age-dependent, tissue-specific somatic mosaicism in a transgenic mouse model of triplet repeat instability. Hum Mol Genet.

[pcbi-0030235-b026] Jung J, Bonini N (2007). CREB-binding protein modulates repeat instability in a *Drosophila* model for polyQ disease. Science.

[pcbi-0030235-b027] Cannella M, Maglione V, Martino T, Simonelli M, Ragona G (2005). New Huntington disease mutation arising from a paternal CAG34 allele showing somatic length variation in serially passaged lymphoblasts. Am J Med Genet B Neuropsychiatr Genet.

[pcbi-0030235-b028] Manley K, Shirley TL, Flaherty L, Messer A (1999). Msh2 deficiency prevents in vivo somatic instability of the CAG repeat in Huntington disease transgenic mice. Nat Genet.

[pcbi-0030235-b029] Goldstein DB, Schlatterer C (1999). Microsatellites: Evolution and applications.

[pcbi-0030235-b030] Brinkmann B, Klintschar M, Neuhuber F, Huhne J, Rolf B (1998). Mutation rate in human microsatellites: Influence of the structure and length of the tandem repeat. Am J Hum Genet.

[pcbi-0030235-b031] Mateo I, Llorca J, Volpini V, Corral J, Berciano J (2004). Expanded GAA repeats and clinical variation in Friedreich's ataxia. Acta Neurol Scand.

[pcbi-0030235-b032] De Michele G, Filla A, Criscuolo C, Scarano V, Cavalcanti F (1998). Determinants of onset age in Friedreich's ataxia. J Neurol.

[pcbi-0030235-b033] Gusella JF, MacDonald ME (2000). Molecular genetics: Unmasking polyglutamine triggers in neurodegenerative disease. Nat Rev Neurosci.

[pcbi-0030235-b034] Rubinsztein DC (2002). Lessons from animal models of Huntington's disease. Trends Genet.

[pcbi-0030235-b035] Menalled LB, Chesselet MF (2002). Mouse models of Huntington's disease. Trends Pharmacol Sci.

[pcbi-0030235-b036] Fischbeck KH, Lieberman A, Bailey CK, Abel A, Merry DE (1999). Androgen receptor mutation in Kennedy's disease. Philos Trans R Soc Lond B Biol Sci.

[pcbi-0030235-b037] Abel A, Walcott J, Woods J, Duda J, Merry DE (2001). Expression of expanded repeat androgen receptor produces neurologic disease in transgenic mice. Hum Mol Genet.

[pcbi-0030235-b038] Lin CH, Tallaksen-Greene S, Chien WM, Cearley JA, Jackson WS (2001). Neurological abnormalities in a knock-in mouse model of Huntington's disease. Hum Mol Genet.

[pcbi-0030235-b039] Chung MY, Ranum LP, Duvick LA, Servadio A, Zoghbi HY (1993). Evidence for a mechanism predisposing to intergenerational CAG repeat instability in spinocerebellar ataxia type I. Nat Genet.

[pcbi-0030235-b040] Choudhry S, Mukerji M, Srivastava AK, Jain S, Brahmachari SK (2001). CAG repeat instability at SCA2 locus: Anchoring CAA interruptions and linked single nucleotide polymorphisms. Hum Mol Genet.

[pcbi-0030235-b041] Matsuyama Z, Izumi Y, Kameyama M, Kawakami H, Nakamura S (1999). The effect of CAT trinucleotide interruptions on the age at onset of spinocerebellar ataxia type 1 (SCA1). J Med Genet.

