# Supplementary material for: A Universal Mechanism Ties Genotype to Phenotype in Trinucleotide Diseases
Source: PLoS Comput Biol. 2007 Nov 23;3(11):e235. doi: 10.1371/journal.pcbi.0030235 (PMC2082501; doi:10.1371/journal.pcbi.0030235)
Supplement: Supplementary file 1 [file pcbi.0030235.sd001.doc]

**Analytical Model**

**Equations for the mean and standard deviation of the allele size distribution as a function of the model parameters and the patient age.**

Here we derive analytical solutions for the mean and standard deviation of the allele size distribution (equations in figure 1 of the paper). Throughout the derivation allele sizes, *i*, are discrete while as time, *t*, is continuous.

We assume a length dependent expansion rate equation *E= (L-I)*R* .

The smallest disease allele in our model is *I+1* as only alleles that exceeds the initial threshold *I* are assumed to mutate.

Let *i(t)=L(t)-(I+1)* be the distance from the first disease allele. (*i*=0 for the first disease allele)

We will first derive *<i(t)>*, the average of the allele distance *i* from the initial threshold *I* and than add *I* to get the average allele size *<L(t)>*

Let *p_i_(t)* be the probability of an allele having a difference of *i* repeats from the first disease allele *(I+1)* time *t*.

The master equation describing the change in *p_i_(t)* according to the model is:

(1) $\frac{\text{dp}_{i}}{\text{dt}}=R i p_{i-1}-R (i+1) p_{i}$ where $i=0\text{.}\text{.}\text{.}\infty$

assuming a linear relation between allele size *i* and expansion rate.

The generating function of the distribution of allele sizes is:

(2)$F(x,t)=\sum_{i=0}^{\infty} p_{i}(t) x^{i}$

differentiating by *x* and setting to *x=1* results in the mean of the allele size distribution:

(3)$\left\langle i(t) \right\rangle=F^{'}(x,t)|_{x=1}=\sum_{i=0}^{\infty} i p_{i}(t) x^{i-1}|_{x=1}$

where ' denotes derivative by *x*. To obtain the temporal dynamics of the mean we will use the following identities;

(4) $\sum_{i=0}^{\infty} i p_{i-1}(t) x^{i}=x^{2} F^{'}(x)+x F(x)$

(5) $\sum_{i=0}^{\infty} (i+1) p_{i}(t) x^{i}=x F^{'}(x)+ F(x)$

using the master equation 1 and equations 2-4 we differentiate *F* by *t*

(6) $\dot{F}(x,t)=\sum_{i=0}^{\infty} \dot{p}_{i}(t) x^{i}=R (x^{2} F^{'}(x,t)+x F(x,t)-x F^{'}(x,t)-F(x,t))=R (x-1) (x F^{'}(x,t)+F(x,t))$where upper dot denotes derivative by time. Differentiating (6) by *x* and setting to *x=1*:

(7) $\overset{}{\left\langle i \right\rangle}=\frac{d\dot{F}(x)}{\text{dx}}|_{x=1}=R(xF^{'}(x)+F(x))|_{x=1}+R(x-1)(2F^{'}(x)+xF^{''}(x))|_{x=1}$

(8) $\overset{}{\left\langle i \right\rangle}=R(\left\langle i \right\rangle+1)$

The solution of (8) is:

(9) $\left\langle i \right\rangle=\text{Ce}^{\text{Rt}}-1$

Let *D* be the difference between the inherited allele size L_0_ and the initial disease threshold *I*, *D= L_0_-I*. Assigning the inherited allele size at t=0 results:

(10) $\left\langle i \right\rangle(t=0)=D\Rightarrow C=D+1$

Substituting (10) into (9)

(11) $\left\langle i \right\rangle(t)=(D+1)e^{\text{Rt}}-1=(L_{0}-I+1)e^{\text{Rt}}-1$

Giving the equation for the mean of allele size from figure 1

(12) $\mu(L_{0},t)=\left\langle L \right\rangle(t)=I-1+(L_{0}-I+1)e^{\text{Rt}}$

To find the variance and standard deviation of the allele size distribution we first find the quantity *y =<i^2^> -<i>* using the generating function *F*. We differentiate equation (7) by *x* to obtain the second derivative, and set *x=1*:

(13) $\frac{d^{2}\dot{F}(x,t)}{\text{dx}^{2}}|_{x=1}=\dot{y}=\left( \overset{}{\left\langle i^{2} \right\rangle-\left\langle i \right\rangle} \right)=4R F^{'}(x)|_{x=1}+2 R F^{''}(x)|_{x=1}$

From (13,11,3)

(14) $\dot{y}=4 R \left\langle i \right\rangle+2R y=4 R (D+1) e^{\text{Rt}}-4R+2 R y$

solving for *y:*

(15) $y(t)=-4R (D+1) e^{\text{Rt}}+2+C_{1} e^{2\text{Rt}}$

(16) $\text{Var}(i)=y+\left\langle i \right\rangle-\left\langle i \right\rangle^{2}=(C-(D+1)^{2})e^{2\text{Rt}}-(D+1)e^{\text{Rt}}$

We assume the variance at birth is Var(t=0)=0 since all alleles have the same inherited size

(17) $C=(D+1)(D+2)$

(18) $\text{Var}(L_{0},t)=\text{Var}(i,t)=(D+1)(e^{2\text{Rt}}-e^{\text{Rt}})$

Leading to the equation for standard deviation from figure 1

(19) $\sigma(L_{0},t)=\sqrt{D+1}\sqrt{e^{2\text{Rt}}-e^{\text{Rt}}}$

The results of the mean and standard deviation were validated using computer simulations and found to be accurate. The variance is not depended on the inherited length but only on *R* and *t*, thus a shorter onset age (which is depended on the number of inherited repeats) implies smaller variability and faster disease progression.

**Deriving equations for the onset and duration based on the inherited allele size and model parameters.**

In order to estimate the time that a critical portion *C=20%* of the allele distribution has expanded beyond the target threshold *T* we assume that the distribution of alleles approaches a normal distribution. This assumption is valid since the size of each allele is a sum of independent expansions. Computer simulations confirm that the estimates based on this assumption give highly accurate results. Under this assumption one can estimate the 80 percentile value according to the equation:

(20)$L_{\text{80}\text{\%}}=\mu(L_{0},t)+0\text{.}\text{085}\sigma(L_{0},t)=I-1+(L_{0}-I+1)e^{\text{Rt}}+0\text{.}\text{85}\sqrt{(L_{0}-I+1)e^{2\text{Rt}}-e^{\text{Rt}}}$

Solving for *t_onset_*. The time when the 80 percentile reaches the target threshold L_80%_=T :

(21) $t_{\text{onset}}=\frac{1}{R}\text{ln}(\frac{\text{400}D(T-I+0\text{.}\text{6388})-\text{340}\sqrt{D(\text{ID}-\text{DT}-2I(T+1)+1+I^{2}+2T+T^{2}-0\text{.}\text{8194}D)}}{D(\text{400}D-\text{289})})$

where D =(L_0_-I+1) (the inherited allele distance from the initial threshold). The time for the 20 percentile to cross the threshold T is:

(22)

$$t_{\text{20}\text{\%}}=\frac{1}{R}\text{ln}(\frac{\text{400}D(T-I+0\text{.}\text{6388})+\text{340}\sqrt{D(\text{ID}-\text{DT}-2I(T+1)+1+I^{2}+2T+T^{2}-0\text{.}\text{8194}D)}}{D(\text{400}D-\text{289})})$$

The disease duration is t_20%_-t_onset_:

(23)

$$t_{\text{duration}}=\frac{1}{R}\text{ln}(\frac{\text{400}D(T-I+0\text{.}\text{6388})+\text{340}\sqrt{D(\text{ID}-\text{DT}-2I(T+1)+1+I^{2}+2T+T^{2}-0\text{.}\text{8194}D)}}{\text{400}D(T-I+0\text{.}\text{6388})-\text{340}\sqrt{D(\text{ID}-\text{DT}-2I(T+1)+1+I^{2}+2T+T^{2}-0\text{.}\text{8194}D)}})$$

Computer simulation validated that the above equation give accurate results to the onset and duration (compared to the simulation results).

**Fitting disease specific parameters:**

We fit equation 21 to clinical data in order to estimate the disease specific parameters. We obtained previously published clinical data for several diseases and used Matlab software (Mathworks) to fit the model parameter using Nelder-Mead simplex direct search least square error. Since the clinical data is not evenly distributed over the range of repeats it is possible that the fitting can be improved by weighted fit or with using a larger clinical data set. We have also conducted sensitivity analysis for the T and R parameters prediction and derived a range in which the goodness of fit (R^2^) is not reduced by more than 5% from the optimal fit. The predictions and the ranges are presented in the table below as well as the correlation coefficient and the number of samples used.

**Fit results summary**

*I*-Initial threshold

*T*-Target threshold

*R*- Rate of mutation per repeat

| Disease | *I* (repeats) | *T* (repeats)  optimal, (range) | *R* (repeats/year/repeat)  optimal, (range) | *n* (data points) | Correlation Coefficient |
| --- | --- | --- | --- | --- | --- |
| Huntington | 37 | 115,(93,170) | 0.05,(0.04,0.06) | 336 | 0.7 |
| SCA1 | 39 | 90,(80,110) | 0.05,(0.04,0.06) | 46 | 0.86 |
| SCA2 | 35 | 59,(55,69) | 0.035,(0.03,0.045) | 19 | 0.89 |
| SCA3 | 61 | 115,(105,125) | 0.04,( 0.035,0.045) | 52 | 0.78 |
| SCA6 | 21 | 51,(44,71) | 0.035,( 0.03,0.045) | 19 | 0.81 |
| SCA7 | 37 | 107,(97,160) | 0.06,(0.055,0.08) | 65 | 0.69 |
| DM1 | 49 | 4400,(3200,6000) | 0.085,(0.07,0.1) | 15 | 0.84 |
| Friedriech Ataxia | 32 | 1450,(1250,1850) | 0.065,(0.055,0.085) | 40 | 0.72 |

Supplementary Figure S1

Model parameter fitting to onset age clinical data sets of various trinucleotide diseases

The target threshold and basal mutation rate parameters fitted to the previously published clinical data of various trinucleotide diseases^1-5^ according to the mechanism dynamics. The ability to achieve accurate fits for the diseases demonstrates that the mechanism can be universal for all diseases.

1.	David, G. et al. Molecular and clinical correlations in autosomal dominant cerebellar ataxia with progressive macular dystrophy (SCA7). *Hum Mol Genet* **7**, 165-70 (1998).

2.	Schols, L., Bauer, P., Schmidt, T., Schulte, T. & Riess, O. Autosomal dominant cerebellar ataxias: clinical features, genetics, and pathogenesis. *Lancet Neurol* **3**, 291-304 (2004).

3.	Novelli, G. et al. (CTG)n triplet mutation and phenotype manifestations in myotonic dystrophy patients. *Biochem Med Metab Biol* **50**, 85-92 (1993).

4.	Perini, G. I. et al. Cognitive impairment and (CTG)n expansion in myotonic dystrophy patients. *Biol Psychiatry* **46**, 425-31 (1999).

5.	Mateo, I. et al. GAA expansion size and age at onset of Friedreich's ataxia. *Neurology* **61**, 274-5 (2003).
